# Case report: Successful treatment of acute generalized pustular psoriasis with multiple comorbidities with oral tacrolimus

**DOI:** 10.3389/fimmu.2024.1354578

**Published:** 2024-03-19

**Authors:** Mingdan Zhao, Fujun Huang, Lei Tang, Xun Zhou, Miao Zhang, Mengxue Liao, Lirong Liu, Mengya Huang

**Affiliations:** ^1^ Department of Dermatology and Cosmetology, Chongqing Hospital of Traditional Chinese Medicine, Chongqing, China; ^2^ College of Traditional Chinese Medicine, Chongqing Medical University, Chongqing, China

**Keywords:** case report, treatment, acute generalized pustular psoriasis, oral, tacrolimus

## Abstract

Acute generalized pustular psoriasis (GPP) is a serious illness. Despite various treatment methods, there is still lack of effective treatment plans for refractory cases with multiple comorbidities. This case report presents a 67-year-old woman with acute GPP, stage 4 chronic kidney disease (CKD), type 2 diabetes, and cardiovascular disease, in whom skin symptom disappearance and kidney function improvement were observed after the use of oral tacrolimus as the sole therapy. This is the first report on the application of tacrolimus in the treatment of acute GPP, especially refractory acute GPP. The successful treatment indicates that there are shared immune pathways between acute GPP and CKD, and the pathways can be interdicted by tacrolimus. Further studies are needed to optimize the therapy to maximize efficacy and minimize toxicity.

## 1 Introduction

Generalized pustular psoriasis (GPP) is a severe disease with reported mortality rates of 2%–16% (1). It characteristically presents as widespread sterile pustules over an erythematous background (2). Thus far, five subtypes have been described. Acute GPP is the most grievous subtype, and it is often accompanied by systemic symptoms, such as fever, general malaise, anorexia, nausea, and myalgias (3). Common treatments for this disease include methotrexate, cyclosporine, acitretin, corticosteroids, biologics, topical therapy, and phototherapy (4). However, some patients are unable to use these therapies due to therapeutic resistance or drug-related contraindications, so another effective and relatively safe treatment option is needed. Here, we present the case of a female patient who had chronic renal failure, type 2 diabetes, and cardiovascular disease, and then developed acute GPP. The patient’s skin symptoms disappeared and kidney function improved without adverse reactions after only oral tacrolimus was administered. Tacrolimus is a commonly used treatment for CKD, but not a standard treatment for acute GPP. To the best of our knowledge, this study is the first attempt to present tacrolimus in acute GPP. We discuss the same immunological pathogenesis between acute GPP and other chronic diseases as well as the role of tacrolimus in treating these conditions based on treatment experience and relevant literature.

**Figure 1:**
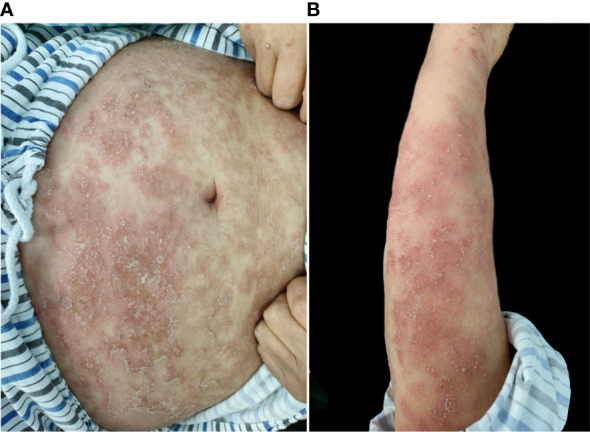
**(A, B)** Initial presentation of abdomen and left upper extremity before treatment.

**Figure 2:**
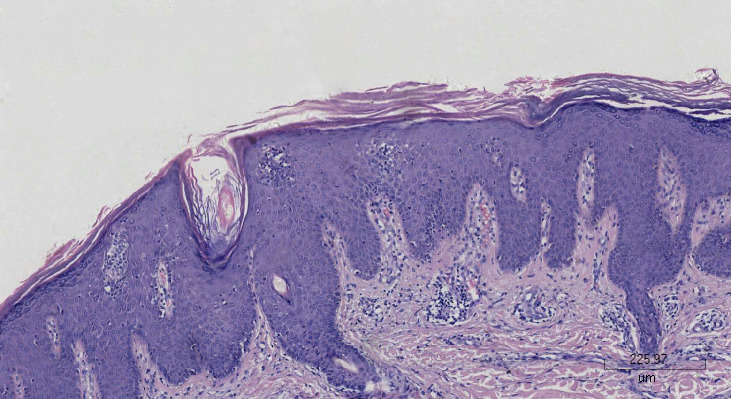
Pathohistological finding of the right upper extremity with hematoxylin–eosin staining.

A 67-year-old woman had psoriasis vulgaris for 1 year. She was treated with topical hormone and narrow-band ultraviolet B phototherapy for a prolonged period of time. Neither of the therapies led to a consistent improvement of cutaneous manifestations, and she developed fever and extensive erythema along with pinpoint pustules for 2 weeks prior to our treatment (**Figures 1A, B**). Her medical history included type 2 diabetes, hypertension, cerebral infarction, and stage 4 CKD. Histopathological examination revealed psoriatic hyperplasia of the epidermis with keratosis, along with Kogoj abscesses in the horny layer. Lymphocytic infiltrate was present in the superficial dermal layer along with dilatation of capillary vessels (**Figure 2**). The patient was diagnosed with acute GPP based on pathological and clinical manifestations.

**Figure 3:**
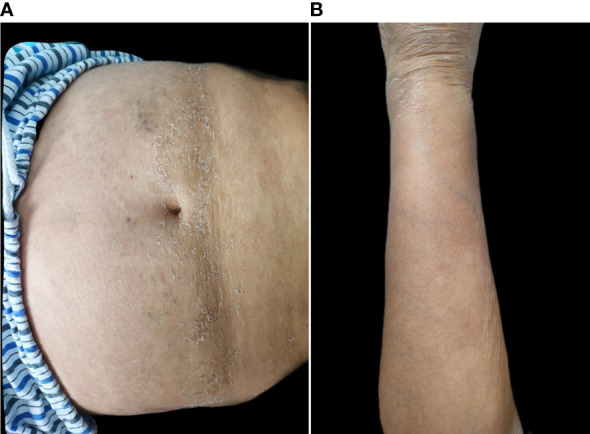
**(A, B)** The lesion of abdomen and left upper extremity after 2 weeks of oral tacrolimus.

Prior to starting systemic treatment, blood tests (blood count, biochemical tests, and inflammatory markers) were performed. We also decided to perform a computer tomography (CT) scan of her chest to exclude an underlying infection, because she had a self-limiting fever episode 2 weeks earlier. The results were unremarkable except for creatinine (247 μmol/L), glomerular filtration (18.3 mL/min/1.73 m^2^), and D-dimers (13.04 mg/L), and the CT scan revealed pulmonary interstitial fibrosis. Acitretin, methotrexate, and cyclosporine were avoided due to serious renal insufficiency. The patient declined systemic corticosteroids due to type 2 diabetes and cardiovascular disease, and the use of topical emollients and steroids was not efficient. With informed consent, the patient received oral tacrolimus at an initial dose of 3 mg/day (0.05 mg/kg/day). The patient was afebrile, and her lesions were almost completely resolved after 2 weeks (**Figures 3A, B**). Creatinine dropped to 213 μmol/L, the glomerular filtration rate was 22 mL/min/1.73 m^2^, and the D-dimers dramatically improved (2.99 mg/L). The patient’s blood pressure and glycemia were well controlled, and no adverse events such as diarrhea, infection, or paresthesia, were observed. The drug dose was reduced by 1 mg every month, and the entire treatment duration was 3 months. No recurrence was observed after 6 months of follow-up.

## 2 Discussion

Acute GPP is an immune-mediated disease that can significantly affect social function and interpersonal relationships (5). GPP is a consequence of genetic susceptibility, and inducements include infections, medications, pregnancy, and environmental factors (6). These inducements lead to the acute activation and infiltration of neutrophils, and the dysregulated inflammatory process has a pivotal part in the development of acute GPP (7). The key cytokines of this pathogenesis include tumor necrosis factor (TNF), interleukin (IL)-1, IL-6, IL-12/23, IL-17A, and IL-36 (8), and they locate on a number of skin cells, such as dendritic cells, monocytes, and keratinocytes. Mitogen-activated protein kinase signaling pathways and their downstream inflammatory pathways are activated when the cytokines express abundantly in skin (9). In addition, CD4+ T cells in blood and skin lesions are activated and intense hyperproliferation leads to the T-cell-mediated inflammation (10). These processes ultimately develop into acute GPP inflammatory skin conditions.

Patients with psoriasis are at high risk of developing other chronic health diseases, such as CKD, cardiovascular disorders, and metabolic syndrome (11, 12). Furthermore, there may be a bidirectional association between psoriasis and these chronic diseases. A GPP study has indicated that hypertension is the most commonly identified comorbidity, followed by diabetes (13). In addition, researchers (14–16) have reported the association of GPP with renal disease, including immunoglobulin A (IgA) nephropathy, and renal amyloidosis. Damasiewicz-Bodzek et al. demonstrated that all peptides, including IgA, increased in patients with psoriasis due to oxidative stress stimulating the immune system (17). There may be a link between psoriasis and IgA nephropathy. Acute phase proteins, including serum amyloid A, accumulated in high amounts in the tissues of patients with GPP, which could increase the risk of developing renal amyloidosis (18). There is a significantly increased risk of incident CKD and end-stage renal disease (ESRD) among patients with psoriasis as compared with the general population (19). Furthermore, patients with GPP may develop acute respiratory distress syndrome, which manifests as noninfectious pneumonitis (20). Renal disease, myocardial infarction history, liver disease, and diabetes mellitus are established as predictors of severe GPP (21). The development of these chronic health diseases may be related to an immune-mediated process. Cytokines, such as IL-36 and TNF-α, activate a broad spectrum of immune and non-immune cells that control various inflammatory processes in the skin, angiocarpy, lung, liver, and kidney (22). T cells are critical drivers of related organ damage by directly promoting inflammation and cytotoxicity or via supporting B-cell differentiation and antibody production (23–25).

Since acute GPP is a potentially life-threatening variant of psoriasis, therapy should be initiated without delay. However, GPP is a multisystem disease that is difficult to treat, so personalized treatment should be selected to improve symptoms and minimize psychological harm. Multiple comorbidities were a limitation for the use of common systemic therapies in our patient. Phototherapy and topical corticosteroids are applied only as adjuvant therapies for psoriatic skin lesions on account of the limited remission rates. Topical vitamin D3 analog is not strongly recommended in the acute-phase treatment of GPP because of skin irritation. Although the literatures suggest that topical tacrolimus is effective in treating GPP (26, 27), application of the therapeutic method is limited because of inadequate skin penetrance. In recent years, there have been increasing biologic agents, including TNF, IL-1, IL-12/23, IL-36, and IL-17 inhibitors, that have been reported in the treatment of acute GPP (28), and patients treated with biologics have demonstrated positive responses. Our patient declined biologics due to the medical expense and relatively weak literature support for patients with stage 4 CKD (29). Granulocyte and monocyte adsorption (GMA) apheresis is also an effective treatment for acute GPP, and it can remove activated granulocytes and monocytes from the patient’s circulation (30). Nevertheless, this therapy relies on an arteriovenous fistula and may lead to a poor dialysis outcome.

Effective and safe therapy was needed in this case. Tacrolimus is a calcineurin inhibitor that prevents calcineurin from dephosphorylating the nuclear factor of activated T cells (31). It results in the blockage of signal transduction pathways in T cells and the inhibition of the synthesis of inflammatory cytokines, such as INF, IL-2, and IL-3 (32). This medicine has been shown to produce successful outcomes in patients with plaque psoriasis with no severe adverse events (33). The average psoriasis area and severity index (PASI) of participants with oral tacrolimus was reduced by 70% (*p* < 0.05) at the end of the 9-week treatment period in a placebo-controlled trial (34). The average PASI had improved by 80.37% (*p* < 0.001) in an open-label prospective study investigating the efficacy and safety of oral tacrolimus treatment for 12 weeks in adults with severe recalcitrant psoriasis (35). However, acute GPP differs from plaque psoriasis in phenotype, genetics, and immunity (36). Fortunately, oral tacrolimus proved to be effective for this patient, and adverse events were not observed. The most frequently reported adverse reactions of tacrolimus are insomnia, tremors, headache, paraesthesia, myalgia, pruritus, and gastrointestinal effects, while significant adverse effects include infection, hypertension, hyperglycemia, hyperkalemia, nephrotoxicity, and neurotoxicity (33). The toxic effects of tacrolimus are not significant due to the low doses and short treatment duration. Furthermore, studies have shown that tacrolimus is a better option than cyclosporine for patients who have comorbidities that include cardiovascular diseases, renal disease, and metabolic syndrome (37, 38). The psoriasis lesions, GFR, creatinine, and D-dimers of this patient improved over time as those diseases shared immune pathways and cytokines. This study supports the results of prior studies on the treatment of renal diseases with tacrolimus, which can induce remissions of creatinine, uric acid, glomerular filtration rate, and proteinuria (39, 40).

## 3 Conclusions

Oral tacrolimus is an effective and relatively safe treatment option for acute GPP patients, especially refractory cases with multiple comorbidities. It can improve systemic inflammation and does not increase cardiovascular burden. This treatment could be a well-tolerated alternative for refractory psoriasis. However, the long-term efficacy and adverse reactions of this therapy are currently unclear. Further studies are necessary to maximize the efficacy and minimize the toxicity of oral tacrolimus.

## Data availability statement

The original contributions presented in the study are included in the article/supplementary material. Further inquiries can be directed to the corresponding author.

## Ethics statement

The studies involving humans were approved by the ethics committee of Chongqing Hospital of Traditional Chinese Medicine. The studies were conducted in accordance with the local legislation and institutional requirements. The participants provided their written informed consent to participate in this study. Written informed consent was obtained from the individual(s) for the publication of any potentially identifiable images or data included in this article.

## Author contributions

MDZ: Resources, Writing – original draft. LT: Writing – original draft, Investigation. XZ: Investigation, Writing – original draft. FH: Methodology, Writing – review & editing. MZ: Validation, Writing – original draft. ML: Writing – original draft, Supervision. LL: Data curation, Writing – review & editing. MH: Writing – review & editing, Writing – original draft.
